# Limited discriminatory performance of the iMCD-IPI in a Western cohort

**DOI:** 10.1093/oncolo/oyag026

**Published:** 2026-03-12

**Authors:** Yoshito Nishimura, Thomas Habermann, Angela Dispenzieri

**Affiliations:** Division of Hematology, Department of Medicine, Mayo Clinic, Rochester, MN, 55905, United States; Division of Hematology, Department of Medicine, Mayo Clinic, Rochester, MN, 55905, United States; Division of Hematology, Department of Medicine, Mayo Clinic, Rochester, MN, 55905, United States

**Keywords:** Castleman disease, iMCD-IPI, TAFRO

## Introduction

Castleman disease (CD) is a rare and heterogeneous lymphoproliferative disorder.^1^ Idiopathic multicentric Castleman disease (iMCD) refers to HHV-8-negative MCD and exhibits a broad clinical spectrum, with some patients having mild constitutional symptoms while others develop life-threatening multi-organ failure due to a cytokine storm.^2^^,^^3^ Given this variability, prognostic models are needed to stratify iMCD patients by risk. The first international prognostic index for iMCD (iMCD-IPI) was published in 2020, derived from a large series of iMCD patients.^4^ The iMCD-IPI was built on 176 patients from the United States (US) and China, and was validated in an additional 197 patients from the international consortium. It identified five adverse risk factors: age >40 years, plasmacytic lymph node histology, hepatosplenomegaly, hemoglobin < 8 g/dL, and pleural effusions. Patients were assigned 1 point for each factor, stratifying them into low (0-1 points), intermediate (2-3 points), or high-risk (4-5 points) categories. In the original report, the model discriminated clinical outcomes well, with high-risk patients having 5-year overall survival (OS) rates of 20% in the high-risk group and 97% in the low-risk group. While the results suggested the iMCD-IPI could guide risk-adapted management, the iMCD-IPI had not been validated in an independent cohort. Additionally, three distinct clinical subtypes have been identified in iMCD since the publication of the data. The aim of this study was to validate the iMCD-IPI using a Western iMCD cohort.

## Methods

We analyzed the full Mayo Clinic Castleman disease database from January 2004 to August 2024. Cases of MCD associated with POEMS syndrome or human herpesvirus 8 were excluded. Clinical and laboratory findings were reviewed to compute the iMCD-IPI as defined initially. All five prognostic variables from the original model were available in our dataset. OS was evaluated from diagnosis to death, and event-free survival (EFS) from diagnosis until an “event” defined as disease progression, start of a new treatment regimen, or death. Patients were censored if they remained alive without progression on initial therapy.

### Definitions

iMCD cases were further categorized as thrombocytopenia, anasarca, fever, reticulin fibrosis/renal dysfunction, and organomegaly (TAFRO) or idiopathic plasmacytic lymphadenopathy (IPL) subtypes if diagnostic criteria for those subtypes were met, or as not otherwise classified (NOS) if not.^3^ Briefly, patients were classified as iMCD-TAFRO if their lymph nodes met histopathologic features of the International iMCD Diagnostic Criteria for CD and had all the following clinical features without another cause: (1) thrombocytopenia (T) defined as pre-treatment nadir platelet level ≤100 × 10^3^/μL; (2) anasarca (A); (3) fever ≥37.5 °C or hyperinflammatory state, defined as CRP ≥ 2.0 mg/dL (F); and (4) organomegaly (O).^5^ Patients were classified as iMCD-IPL if they met al. the following criteria: (1) polyclonal hypergammaglobulinemia (γ-globulin > 4.0 g/dL or serum IgG level >3500 mg/dL), (2) multicentric lymphadenopathy, (3) an absence of definite autoimmune disease, and (4) normal germinal centers and a sheet-like infiltration of polyclonal plasma cells in the lymph node.^6^^,^^7^

### Data and statistical analyses

Time-to-event outcomes (OS, EFS) were estimated using the Kaplan–Meier method, and survival curves were compared by the log-rank test. OS was a primary outcome, and EFS was an exploratory secondary outcome. Further, exploratory subgroup analyses of IPI discrimination within iMCD subtypes and pre- vs post-IL-6 blockade eras (before and after 2014) were also performed. For survival and logistic regression analyses, a *P*-value of <.05 was considered statistically significant. All statistical analyses were performed using JMP Ver. 15.1.

## Results

### Patient cohort

Fifty-one iMCD cases were included in the analysis. Among the 51 patients, 16 had TAFRO, nine had IPL, and 26 had NOS. The median age of iMCD patients was in 43 (95% CI: 34-57), and 80% of the cohort were White. 61% had plasma cell (PC) type histology, and 29% had hyaline vascular or hypervascular (HV) type, with the remaining 10% having mixed histology.

### Application of iMCD-IPI

**Figure 1.**
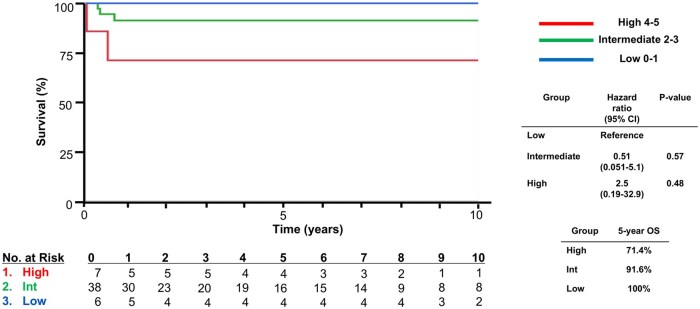
Overall survival of iMCD based on iMCD-IPI. iMCD, idiopathic multicentric Castleman disease.

**Figure 2.**
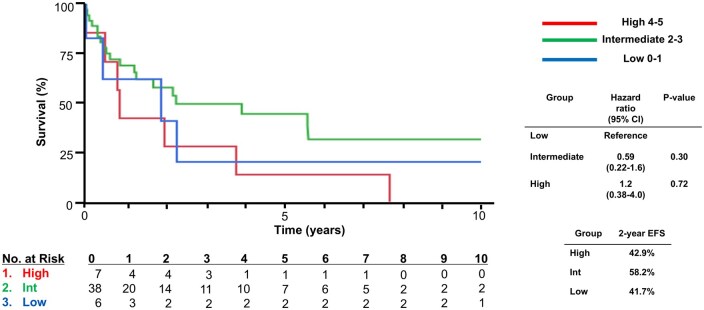
Event-free survival of iMCD based on iMCD-IPI. iMCD, idiopathic multicentric Castleman disease.

Based on iMCD-IPI risk stratification, six (11.7%), 38 (74.5%), and seven (13.7%) were classified as low-risk, intermediate-risk, and high-risk, respectively. OS based on iMCD-IPI risk stratification in our cohort is shown in Figure 1. No significant difference was found among the low-, intermediate-, and high-risk disease groups. Hazard ratio (HR) in the high-risk group was 2.5 (0.19-32.9) compared to the low-risk group (log-rank *P*-value = .48). There was also no difference in the exploratory EFS analyses between the low-, intermediate-, and high-risk disease groups, with HRs of 0.59 (0.22-1.6) in the intermediate and 1.2 (0.38-4.0) in the high-risk groups compared to the low-risk group as a reference (Figure 2).

To complement Kaplan–Meier comparisons, we evaluated the C-index from including iMCD-IPI risk group as the predictor. The C-index for OS was 0.64. We also estimated exploratory time-point AUC values at five years (AUC 5-year: 0.76). For calibration, we compared observed OS in our cohort at five years with survival estimates reported in the original iMCD-IPI derivation/validation study (low: 97.4%; intermediate: 72.2%; high: 20.0%). Observed 5-year OS in our cohort was 100% (low), 92.1% (intermediate), and 71.4% (high).

### Exploratory subgroup analyses

Given evolving subtype definitions and changes in practice, we performed exploratory stratified analyses by (1) subtype (TAFRO, IPL, and NOS) and (2) treatment era (pre–anti–IL-6 vs. post–anti–IL-6 availability). Four, 11, and one had high-, intermediate-, and low-risk disease in the TAFRO subtype, respectively (Figure S1A—see online supplementary material for a color version of this figure). Among IPL subtype patients, one had high-risk disease while the other eight had intermediate-risk disease (Figure S1B—see online supplementary material for a color version of this figure). For NOS, two, 19, and five had high, intermediate, and low risk patients, respectively (Figure S1C—see online supplementary material for a color version of this figure). Among the pre-anti-IL-6 cohort (Figure S2A—see online supplementary material for a color version of this figure), there were two, 12, and two high, intermediate, and low risk patients, respectively. For the post-anti-IL-6 cohort (Figure S2B—see online supplementary material for a color version of this figure), there were five, 26, and four high-, intermediate-, and low-risk patients, respectively. In the exploratory subgroup analyses, HRs could not be calculated due to an unstable model.

## Discussion

This application of the iMCD-IPI to the Western cohort did not clearly demonstrate discrimination for OS. While these findings should be interpreted with caution due to small sample size, several factors may explain reduced discriminatory performance relative to the original iMCD-IPI. First, the original iMCD-IPI was derived in an earlier diagnostic and therapeutic context and included relatively few high-risk patients, raising the possibility of statistical overfitting. Also, the iMCD-IPI treated PC type histology as an adverse prognostic factor based on univariate analysis in the original study that associated PC type histology with worse OS. However, there was no statistical significance in the multivariate analysis, and emerging data suggest that PC type histology in iMCD, one of the characteristics of iMCD-IPL, may be associated with favorable clinical courses and responses to anti-interleukin-6 (IL-6) therapy. iMCD-IPL, previously considered as a part of NOS, is characterized by chronic progressive symptoms and may be well-defined by a combination of histopathology findings, including the PC type histology.^6–8^ In an era or setting where those iMCD subtypes were not yet recognized, and IL-6 blockade was not readily available, such patients might have had a poor prognosis, making PC type histology appear to be a predictive variable.

Further, the iMCD-IPI was derived from the patient cohort predominantly observed or treated with chemotherapy and treatment-era effects are likely important. The increasing use of guideline-concordant therapy, particularly anti–IL-6 monoclonal antibody, may improve outcomes across risk strata and narrow absolute survival differences. In exploratory analyses stratified by subtype and era, iMCD-IPI separation remained limited; however, these analyses were underpowered and are presented primarily to contextualize contemporary practice rather than to draw firm conclusions. Notably, in the original discovery cohort, only nine (5.1%) and three (1.7%) received siltuximab, an anti-IL-6 monoclonal antibody in use for iMCD in the US,^9^ and tocilizumab as either first-line or second-line therapies, respectively. In our cohort, which spans from 2004 to 2024, 43% received siltuximab as either first- or second-line treatment, and 59% received rituximab by the second line. As treatment following the recent iMCD guidelines, which recommend siltuximab for first-line settings, has been shown to improve outcomes,^10^ the changes in diagnostic and treatment paradigms highlight that prognostic models may need recalibration over time and across healthcare settings.

The study has limitations. First, since we did not perform a central review, we may not be able to clearly state that the plasmacytic variant, old nomenclature used in the original article and the PC type histology describes same histopathology. Second, although OS was not significantly different based on iMCD-IPI, given the limited sample size, it might be underpowered to detect the difference. Finally, EFS is sensitive to evolving treatment patterns and thresholds for changing therapy over two decades. Accordingly, OS was emphasized as the primary endpoint in this revision. In conclusion, in this Western cohort, the iMCD-IPI demonstrated limited discriminatory performance. Future prognostic tools for iMCD may require multicenter collaboration, adequate event counts, and incorporation of modern subtype definitions and contemporary treatment patterns to ensure clinical utility in this rare disease.

## Supplementary Material

oyag026_Supplementary_Data
