# Supplementary figures and images for: Limited discriminatory performance of the iMCD-IPI in a Western cohort
Source: Oncologist. 2026 Mar 12;31(4):oyag026. doi: 10.1093/oncolo/oyag026 (PMC12995427; doi:10.1093/oncolo/oyag026)

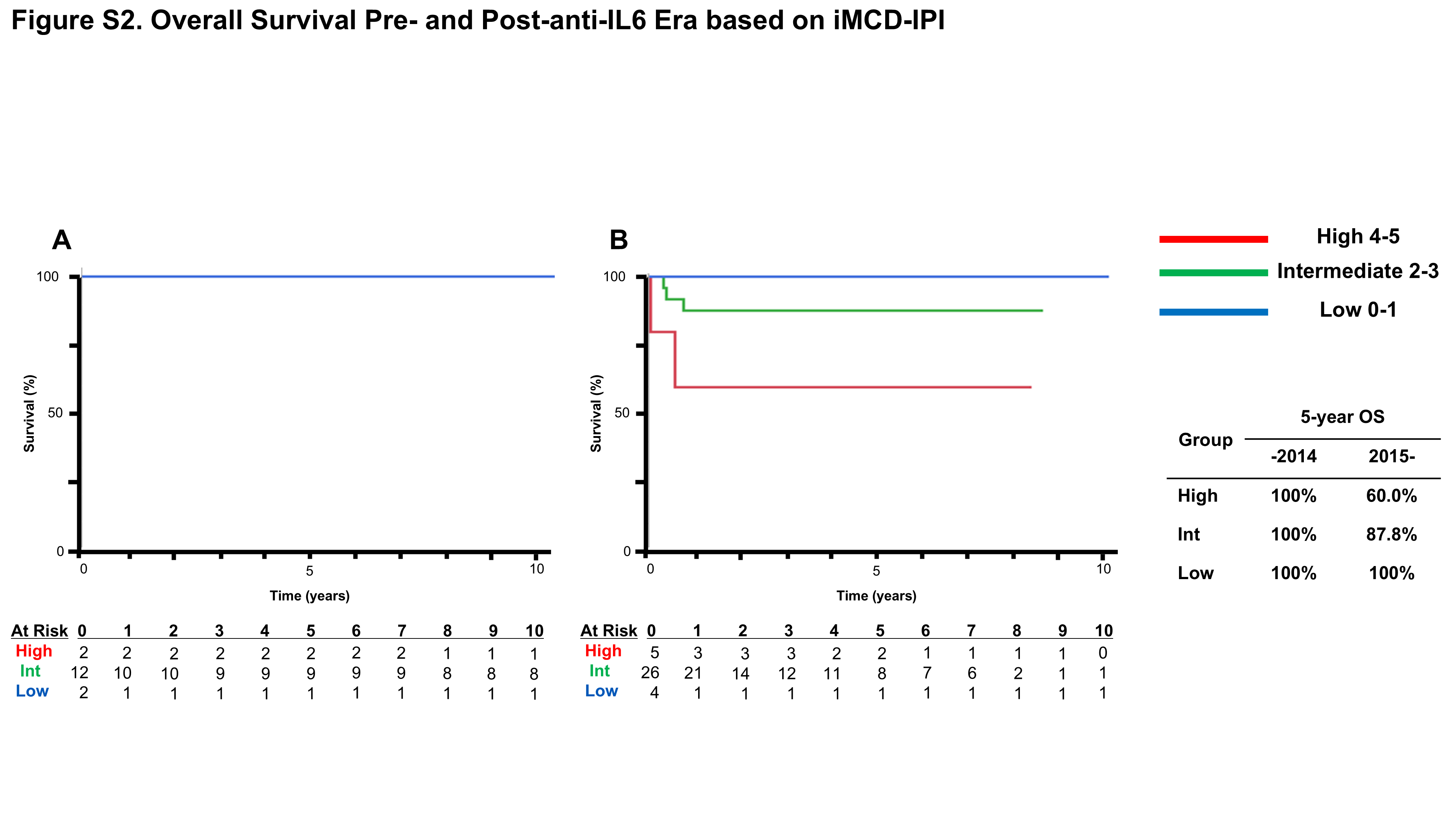

Supplement: oyag026_Supplementary_Data [file oyag026_supplementary_data.zip › Figure S2.TIF]

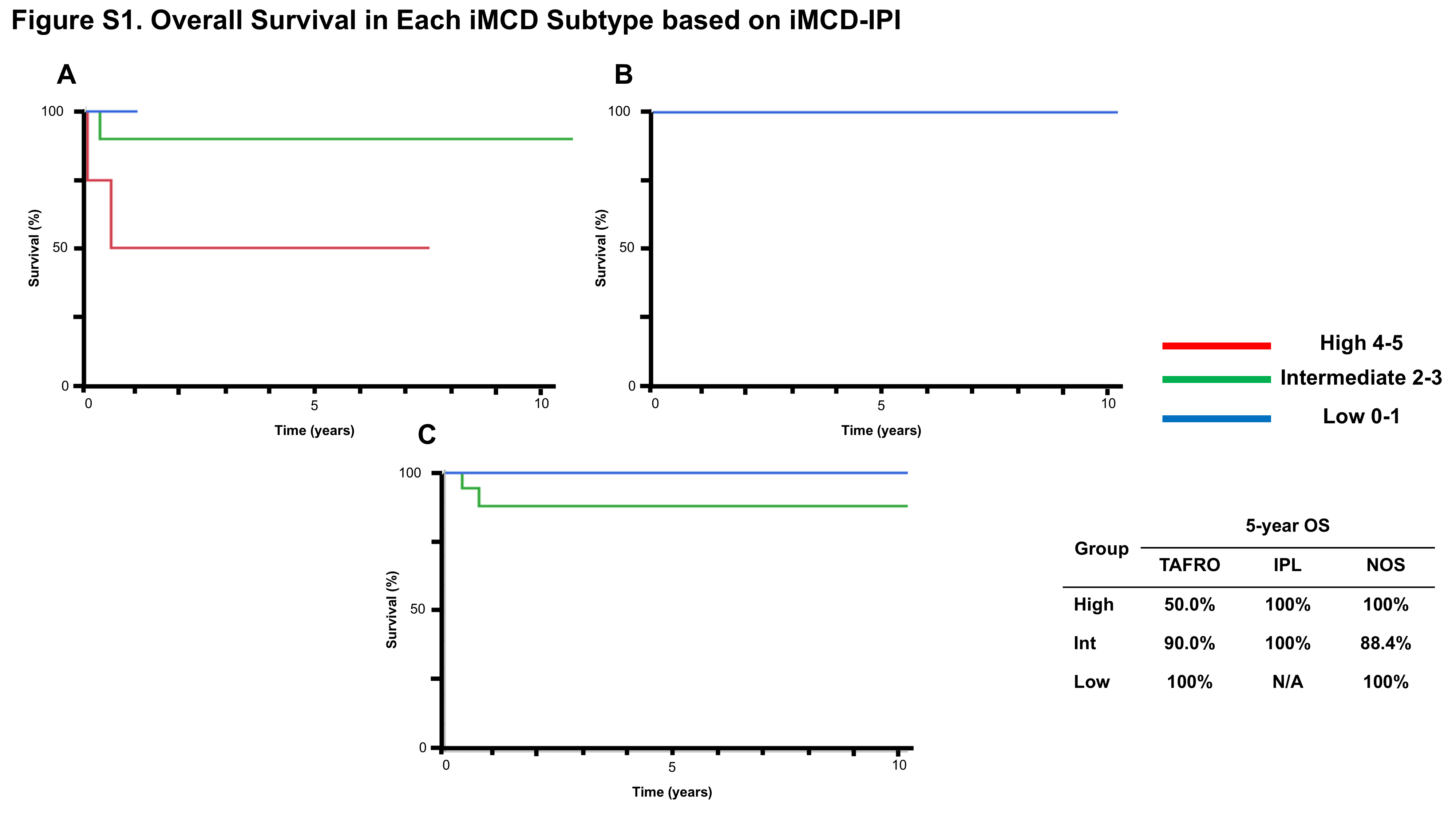

Supplement: oyag026_Supplementary_Data [file oyag026_supplementary_data.zip › Figure S1.TIF]
